# Prevalence of pathogenic free-living amoeba and other protozoa in natural and communal piped tap water from Queen Elizabeth protected area, Uganda

**DOI:** 10.1186/s40249-016-0162-5

**Published:** 2016-08-03

**Authors:** Celsus Sente, Joseph Erume, Irene Naigaga, Julius Mulindwa, Sylvester Ochwo, Phillip Kimuda Magambo, Benigna Gabriela Namara, Charles Drago Kato, George Sebyatika, Kevin Muwonge, Michael Ocaido

**Affiliations:** 1 Department of Wildlife and Aquatic Animal Resources (WAAR), School of Veterinary Medicine and Animal Resources (SVAR), College of Veterinary Medicine, Animal Resources and Biosecurity (COVAB), Makerere University, P.O.Box 7062, Kampala, Uganda; 2 Department of Biomolecular Resources and Biolab Sciences, School of Bio-security, Biotechnical and Laboratory Sciences (SBLS), College of Veterinary Medicine, Animal Resources and Biosecurity (COVAB), Makerere University, P.O.Box 7062, Kampala, Uganda; 3 Department of Biochemistry and Sports Science (BSS), College of Natural Sciences (CONAS), Makerere University, P.O. Box 7062, Kampala, Uganda; 4 Medical Research Council (MRC)/Uganda Virus Research Institute (UVRI), Research Unit on AIDS, P.O.Box 49, Entebbe, Uganda; 5 Department of Molecular Biology, Vrije Universiteit, Pleinlaan 21050, Brussels, Belgium

**Keywords:** Prevalence, Free-living, Protozoa, Water, Queen Elizabeth, Uganda

## Abstract

**Background:**

Pathogenic water dwelling protozoa such as *Acanthamoeba* spp., *Hartmannella* spp., *Naegleria* spp., *Cryptosporidium* spp. *and Giardia* spp. are often responsible for devastating illnesses especially in children and immunocompromised individuals, yet their presence and prevalence in certain environment in sub-Saharan Africa is still unknown to most researchers, public health officials and medical practitioners. The objective of this study was to establish the presence and prevalence of pathogenic free-living amoeba (FLA), *Cryptosporidium* and *Giardia* in Queen Elizabeth Protected Area (QEPA).

**Methods:**

Samples were collected from communal taps and natural water sites in QEPA. Physical water parameters were measured in situ. The samples were processed to detect the presence of FLA trophozoites by xenic cultivation, *Cryptosporidium* oocysts by Ziehl-Neelsen stain and *Giardia* cysts by Zinc Sulphate floatation technique. Parasites were observed microscopically, identified, counted and recorded. For FLA, genomic DNA was extracted for amplification and sequencing.

**Results:**

Both natural and tap water sources were contaminated with FLA, *Cryptosporidium* spp. and *Giardia* spp. All protozoan parasites were more abundant in the colder rainy season except for *Harmannella* spp. and *Naegleria* spp. which occurred more in the warmer months. The prevalence of all parasites was higher in tap water than in natural water samples. There was a strong negative correlation between the presence of *Acanthamoeba* spp., *Hartmannella* spp., *Cryptosporidium* spp. and *Giardia* spp. with Dissolved Oxygen (DO) (*P* < 0.05). The presence of *Cryptosporidium* spp. showed a significant positive correlation (*P* < 0.05) with conductivity, pH and Total Dissolved Solids (TDS); whereas the presence of *Giardia* spp. had only a strong positive correlation with TDS. Molecular genotyping of FLA produced 7 *Acanthamoeba,* 5 *Echinamoeba*, 2 *Hartmannella*, 1 *Bodomorpha*, 1 *Nuclearia* and 1 *Cercomonas* partial sequences.

**Conclusions:**

All water collection sites were found to be contaminated with pathogenic protozoa that could possibly be the cause of a number of silent morbidities and mortalities among rural households in QEPA. This implies that water used by communities in QEPA is of poor quality and predisposes them to a variety of protozoan infections including the FLA whose public health importance was never reported, thus necessitating adoption of proper water safety measures.

**Electronic supplementary material:**

The online version of this article (doi:10.1186/s40249-016-0162-5) contains supplementary material, which is available to authorized users.

## Multilingual abstracts

Please see Additional file 1 for translations of the abstract into the five official working languages of the United Nations.

## Background

The factors that contribute to the emergence and re-emergence of infectious diseases originate from changes in the ecosystems; such as ecosystem variations of natural or anthropogenic origin with climatic and geographic influences on pathogens and vectors, or changes in the microbes themselves, or their host spectrum [1, 2]. Such changes in the ecosystem could be the cause of the emergence of infections such as Zika, Ebola, Marburg, Influenza and West Nile viruses as well as protozoan and other parasitic infections. Pathogenic waterborne protozoan parasites such as *Acanthamoeba, Hartmannella, Naegleria, Cryptosporidium* and *Giardia* are typically associated with poor and often marginalised communities, as observed mostly by studies done in America, Asia, Europe and the Middle East [3–5]. Lack of adequate information about waterborne parasites in sub-Saharan Africa especially on FLA has compelled the present study.

In many rural African households, untreated water is used for various purposes such as bathing, cooking, drinking and swimming, often exposing them to waterborne parasitic infections [3, 5]. More than 300 million people in sub-Saharan Africa have poor access to safe water, predisposing them to a number of infections directly or indirectly, the commonest being diarrhoeal infections which have been reported to be the second leading cause of death in children under 5 years [3, 6]. The commonly studied waterborne pathogens in poor rural households are those associated with typhoid, dysentery, cholera, hepatitis E and rotavirus in children and immunocompromised individuals [7–10]. Other waterborne parasites such as pathogenic free-living amoeba (FLA) that may cause severe health effects in humans and animals have not been widely studied in developing countries. However, they have been studied more in developed countries and are known to be associated with severe infections in humans [5]. In sub-Saharan Africa, due to lack of information, more studies are needed to establish the health importance of FLA [5].

Pathogenic FLA such as *Acanthamoeba* spp., *Hartmanella vermiformis, Balamuthia mandrillaris, Naegleria* spp. and *Vahlkampfia avara* are aerobic eukaryotic protists that can potentially cause infections in humans and animals [7]. They have been implicated in infections of the central nervous system, eye, nose and skin. Most pathogenic FLA are known to facilitate intracellular multiplication of *Legionella pneumophila*, *Vibrio cholerae*, *Bacillus anthracis* and *Mycobacterium tuberculosis* which are responsible for legionellosis, cholera, anthrax and tuberculosis, respectively [11, 12]. *Acanthamoeba* has been reported as a causative agent of granulomatous amoebic encephalitis (GAE), a fatal disease of the CNS and amoebic keratitis (AK), a painful sight-threatening disease of the eyes [11]. It has also been associated with cutaneous lesions and sinusitis in HIV/AIDS patients and other immunocompromised individuals [13]. A case of mixed keratitis infection has been reported due to *Acanthamoeba* spp. and thermotolerant *H. vermiformis* [14]. *Hartmannella* like *Acanthamoeba* is also a host of a number of human bacterial organisms such as *Legionella pneumophila* and *Pseudomonas aeruginosa* [15, 16]. *Naegleria fowleri*, *N. australiensis* and *N. italica* are associated with acute, fulminant, necrotizing and hemorrhagic primary amoebic meningoencephalitis (PAM) that often causes death in children and adults with a history of recent contact with fresh water [7]. Other FLA such as *Cercozoa* (*Cercomonadida, Thaumatomonadida, Euglyphida, Desmothoracida, Gromiidae, Phaeodarea*), *Platymoeba* and *Echinamoeba* (*E. thermarum*, *E. exundans*) have not yet been reported as pathogenic, but with ecosystem changes, climatic changes, geographic influences and mutations, they could as well cause infections in the near future.

Over the past decade, *Cryptosporidium* and *Giardia* have emerged as major waterborne pathogens [17] whose transmission occurs as a result of water contamination with animal and human faeces. These infections are attributed to poor hygiene followed by lack of clean potable water resulting in cryptosporidiosis and giardiasis which are characterised by vomiting, chills, headache, fever, profuse diarrhoea, abdominal pain and cramping [18, 19]. *C. parvum* and *G. lamblia* can cause severe clinical infections in young individuals [20]. Infections caused by *Cryptosporidium* and *Giardia* pose a significant environmental and public health concern because of their tremendous ability to be transmitted from animal to animal and from animal to humans [20].

Free-living amoeba, *Cryptosporidium* and *Giardia* are parasites commonly found in natural water resources and piped water systems, however not much is known about their occurrence, prevalence and distribution in both rural and urban communities in Uganda.

## Methods

### Study area

**Fig. 1:**
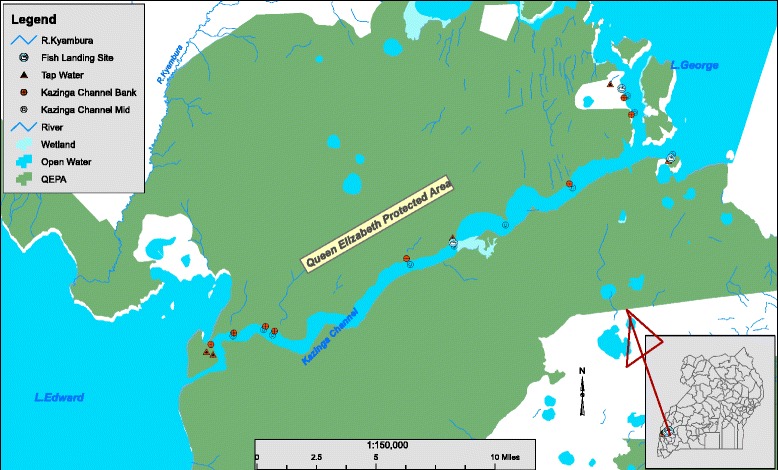
Map of the study area

The study was conducted in Queen Elizabeth Protected Area (QEPA), Uganda (Fig. 1). It is located at 00 12S, 30 00E (Latitude: 0.2000; Longitude: 30.0000) and is 1 978 sq. km in size. This Protected Area (PA) harbours Lakes George and Edward joined by the 40 km long Kazinga Channel. QEPA is a UNESCO ‘Man and Biosphere Reserve’ with 11 village enclaves, all with a fast growing population of humans whose main economic activities are fishing and livestock production. Their source of water is direct natural water from Kazinga channel, River Kyambura, Lake George and Lake Edward or untreated piped tap water channelled directly from the natural sources and stored in reservoir tanks before it is supplied to them.

### Sample collection, storage and transportation

The water sources considered were those from natural water sources and tap water systems. The sampling sites were purposively selected based on their benefit, convenience and importance to public health. They were based on certain landmarks that included the following: (1) along River Kyambura (R. Kyambura), (2) Kazinga channel banks (KCB), (3) Kazinga mid channel (KMC) (water in the middle of the channel), (4) Fish landing sites (FLS), and (5) Community piped tap water. Sampling was done within 1 year in the months of November, January, March, May, July and September. Important physical water parameters including; Dissolved Oxygen (DO) (mg/L), surface water temperature (°C), conductivity (μS/m), pH and Total Dissolved Solids (TDS)(g/L) were determined in situ using a Multi-parameter water sensor (Greenspan, USA). A total number of 408 (324 natural water and 84 tap water) water samples were collected using 50 ml sterile polypropylene falcon tubes (Falcon® Centrifuge Tubes, Discovery Labware, USA). All samples were stored at room temperature and transported to the Makerere University parasitology laboratory within 48 h.

### Laboratory methods

Three methods were used for parasite detection. Culture in non-nutritive media for FLA, Modified Ziehl-Neelsen staining for *Cryptosporidium* and the Zinc Sulphate flotation technique for *Giardia* cysts. For the FLA positive samples, DNA extraction, amplification and sequencing were carried out.

#### Sample processing and growth in xenic cultures

The non-nutritive medium (Page Amoeba Saline solution of 2.5 mM NaCl, 1 mM KH_2_PO_4_, 0.5 mM Na_2_HPO_4_, 40 mM CaCl_2_ and 20 mM MgSO_4_) was seeded with 0.1 ml of a heat inactivated 48-h culture of *E. coli BL2* [21]*.* Water samples collected in the 50 ml tubes were centrifuged at 1 000 × *g* for 15 min and supernatant poured off to expose the pellets. Using sterile Pasteur pipettes, the pellets were removed from all the tubes and carefully spread on pre-seeded NNA-EI agar plates. The plates were incubated at 32 °C overnight. The following day each plate was sealed with a plastic film and incubated upside down at 32 °C up to 7 days. After 3 days of incubation, the plates were monitored for detection of FLA trophozoites until the 7^th^ day using an inverted microscope (Motic® AE2000 Binocular, TED PELLA Inc. USA). The number of FLA trophozoites were counted using a haemocytometer (MicrobeHunter, Germany) and recorded.

#### Modified Ziehl-Neelsen carbol fuchsin staining for cryptosporidium

To maximise recovery of oocysts, the samples were centrifuged at 1 000 × *g* for 10 min to concentrate the oocysts. A modified Ziehl-Neelsen’s (ZN) carbol-fuchsin stain [18] was used to identify the oocysts of *Cryptosporidium* spp. A few drops of water were placed on a slide and stained with ZN-carbol fuchsin stain for 2 min, rinsed with tap water, followed by rinsing with acid alcohol (3 % hydrochloric acid in 70 % ethanol) and subsequently with tap water again. The product was counterstained with Brilliant Green for 2 min before rinsing it with tap water. This was followed by air-drying the slide before viewing under a microscope.

### Zinc sulphate floatation technique for *Giardia*

Zinc Sulphate floatation technique [22] was used to identify Giardia cysts. To maximise recovery of cysts, each water sample was concentrated by centrifugation at 1 000 × *g* for 1 min. The supernatant was poured off leaving a small pellet. A test tube was filled with Zinc Sulphate solution (33 % w/v, Specific gravity 1.18-1.2). An estimated 1-2 ml of the water was added to the test tube and stirred. The test tube was then filled to the brim with Zinc Sulphate solution. A grease-free cover slip was put on the full to the brim test tube and left for 15 min to give time for the cysts to float. After 15 min, the cover slip was carefully lifted off the test tube, placed face down on a microscope slide and viewed under a microscope to identify the cysts.

#### Identification and counting parasites at genus level

To determine the genus of each protozoan parasite, its movement and structural properties were examined [23]. Representatives trophozoites/(oo)cysts of FLA, *Cryptosporidium* and *Giardia* were counted using a haemocytometer (Bright-Line™^,^ Sigma-Aldrich Co. LLC, USA) and recorded.

#### Deoxyribonucleic acid (DNA) extraction

Genomic DNA was extracted from only FLA culture positive plates by chemical lysis and purification with phenol/chloroform/isoamyl alcohol extraction method [24]. This involved adding 500 μl of STE buffer (0.1 M NaCl, 1 mM EDTA, 10 mM Trischloride, pH 8, 1 % SDS) and 10 μl proteinase K (10 mg/ml) directly to each sample in Eppendorf tubes. The samples were incubated at 56 °C for one hour and then cooled before phenol extraction was started. Equal volumes of phenol-chloroform (521 μl) were added to the samples, mixed by vortexing and centrifuged at maximum speed (13 200 rpm) for 10 min. The aqueous layers from each tube were recovered and transferred to new Eppendorf tubes. This step was repeated to make two phenol-chloroform extractions. The aqueous layers were subjected to another chloroform extraction, recovered by centrifugation and transferred to new Eppendorf tubes, after which 1 000 μl of absolute alcohol (96-100 %) was added to each sample. The samples were then put in a freezer at -80 °C for precipitation overnight. The next day, samples were removed from the freezer and centrifuged at 13 200 rpm for 30 min. Absolute alcohol was poured off. The pellet in each tube was then washed with 1 000 μl of 70 % alcohol, centrifuged at 13 200 rpm for 15 min and alcohol poured off to expose the pellet. Finally, the pellet was air dried and dissolved in 50 μl of TE buffer.

#### DNA amplification

Amplification of the partial 18S ribosomal DNA (18S rDNA) gene from FLA was performed using primer pairs JDP1/JDP2 and CRN5/1137 [25, 26]. The JDP1/JDP2 primer was specific to *Acanthamoeba* organisms whereas CRN5/1137 obtained amplimers from any eukaryote, aiding amplification of the 18S Ribosomal DNA gene from *Hartmanella, Cercozoa, Bodomorpha*, *Echinamoeba* and several other groups of FLA.

Amplification reactions were performed using a DreamTaq PCR kit (Thermoscientific DreamTaq, USA). We used a 25 μl reaction volume containing 12.5 μl DreamTaq Green PCR Master Mix (2X), 0.5 μM forward primer, 0.5 μM reverse primers, 9 μl nuclease free water and 2.5 μl DNA template (50 pg concentration). The PCR was done under the following conditions: Initial denaturation at 94 °C for 3 min then 35 cycles with denaturation at 94 °C for 30 s, annealing at 55 °C for 30 s, extension at 72 °C for 30 s and a final extension at 72 °C for 5 min. A sample of 5 μl of each PCR reaction was screened for successful amplification on a 2.5 % (W/V) agarose gel stained with ethidium bromide and run against 1 kb DNA ladder (Finnzymes, Finland). Electrophoresis was performed at 100 V of current and buffer used was 1 × TAE containing 0.5 μg/ml of ethidium bromide. Once enough electrophoretic separation was realised, the agarose gel was observed using a UV gel documentation system (Wagtec, UK). The gel images were captured and a soft copy stored.

#### Nucleic acid sequencing and analysis

The samples that showed the strongest positive bands between 400-600 bp with JDP1/JDP2, and 1-1 475 bp with CRN5/1137) were extracted from the gel and the DNA was purified using QIAquick gel extraction kit (Qiagen Inc. Sample and Assay Technologies, Netherlands). The 18S rDNA segment from each of the FLA isolates was subjected to cycle sequencing using Dyenamic Terminator Cycle sequencing kit with JDP1/JDP2 and CRN5/1137 as sequencing primers [26]. The sequencing included 2 μl of PCR product, 5 × BigDye Buffer, and 2 pmol primer. Sequencing was done in 30 cycles with step 1 at 94 °C for 30 s, step 2 at 55 °C for 15 s and step 3 at 65 °C for 4 min. The sequence files were checked for quality and base trimming carried out using the Seqbuilder software (Dnastar, USA). For each of the nucleotide query sequences, a search for homologues in the NCBI database was carried out using the blastn tool. Homologues with query coverage >75 %, identity >70 % and low E values were considered. The molecular phylogenetic analysis was then completed by using the Maximum Likelihood Method in MEGA6 [27].

### Statistical analysis

Data was analysed using IBM SPSS version 22. Numerical variables were summarised using mean and standard error of the mean (SEM). Univariate analysis to compare the prevalence of parasites across sampling sites was done using cross-tabulation with a Chi-square or Fisher’s exact test. Variables with a *P*-value of ≤ 0.05 were taken to be significant. Correlation analysis between environmental variables and waterborne parasite presence was done using Pearson correlation coefficient (r), a *P*-value of ≤0.05 was considered statistically significant.

## Results

### Prevalence of the organisms

**Fig. 2:**
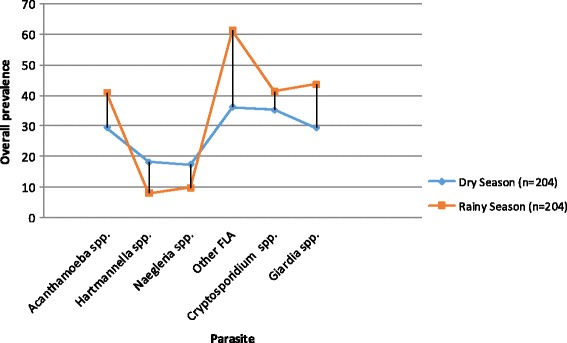
Overall seasonal prevalence of the protozoan parasites

The water samples were collected during cold rainy (November, March & July) and cool dry (January, May & September) seasons. Overall, protozoan parasite prevalence was higher during the rainy season except for *Hartmannella* and *Naegleria* spp. that were higher in the dry season (Fig. 2). The prevalence and means (SEM) of the parasites from different sources are shown in Table 1. Both natural and tap water sources were contaminated with FLA, *Cryptosporidium* spp. and *Giardia* spp. The prevalence of all the parasites was higher in tap water, whereas as mean (SEM) was higher in natural water. The prevalence and mean of the parasites from the natural water sites were highest at KCB. The number of organisms isolated was significantly influenced by the sampling site.

**Table 1:** Waterborne parasite prevalence (%) and mean (SEM) per water source

Parasite		Sources					
		Tap water (*n* = 84)	Natural water (*n* = 324)	R. Kyambura (*n* = 48)	KCB (*n* = 84)	FLS (*n* = 60)	KCM (*n* = 132)
*Acanthamoeba* spp.	(+)(%)	36(43)	107(33)	19(39.6)	51(60.7)	30(50)	7(5.3)
	Mean(±SEM)	2.26 ± 0.4	8.92 ± 1.6	2.23 ± 0.53	3.44 ± 0.49	3.08 ± 0.53	0.17 ± 0.08
*Hartmannella* spp.	(+)(%)	19(22.6)	73(22.5)	11(22.9)	26(43.3)	29(34.5)	7 (5.3)
	Mean(±SEM)	1.20 ± 0.15	5.93 ± 0.92	1.19 ± 0.33	2.60 ± 0.37	2.4 ± 0.22	0.12 ± 0.1
*Naegleria* spp.	(+)(%)	12(14.3)	43(13.3)	17(20.2)	12(25)	6(10)	8(6.1)
	Mean(±SEM)	0.5 ± 0.15	1.58 ± 0.6	0.5 ± 0.15	0.51 ± 0.14	0.43 ± 0.2	0.14 ± 0.1
Other FLA	(+)(%)	29(34.5)	77(23.8)	29(34.5)	19(39.6)	16(26.7)	13(9.8)
	Mean(±SEM)	2.95 ± 0.40	6.2 ± 1.10	0.74 ± 0.16	2.95 ± 0.40	0.35 ± 0.11	0.5 ± 0.9
*Cryptosporidium* spp.	(+)(%)	34(40)	22(26.2)	14(29.2)	62(73.8)	28(46.7)	30(22.7)
	Mean(±SEM)	45.24 ± 9.34	416.77 ± 56.88	37.50 ± 9.24	223.81 ± 21.33	101.67 ± 16.03	53.79 ± 10.28
*Giardia* spp.	(+)(%)	32(38.1)	117(36.1)	12(25)	32(53.3)	38(45.2)	35(26.5)
	Mean(±SEM)	83.33 ± 14.15	425.52 ± 69.82	43.75 ± 11.87	152.38 ± 21.39	148.33 ± 22.61	81.06 ± 13.95

### Physical parameters

The means (SEM) of physical parameters are presented in Table 2. The mean DO (mg/L) from highest to lowest was as follows; KCM (4.56 ± 0.26), Tap water (4.08 ± 0.27), River Kyambura (4.03 ± 0.15), FLS (1.84 ± 0.21) and KCB (1.74 ± 0.15). All water sources had an alkaline condition (pH 7.99 ± 0.11- pH 9.31 ± 0.04) at 21.43 ± 0.14 (°C) - 5.76 ± 20 (°C). The mean TDS (g/L) was lowest at KCM with no significant difference at all the other points. Conductivity (μS/m) did not exhibit much difference.

**Table 2:** Physical parameters of different water sources

Water source	DO(mg/L)	pH	Temp (°C)	Conductivity (μS/m)	TDS(g/L)
R. Kyambura	4.03 ± 0.15	7.99 ± 0.11	21.43 ± 0.14	250.98 ± 3.02	113.12 ± 6.42
KCB	1.74 ± 0.15	9.31 ± 0.04	25.63 ± 24	273.71 ± 9.26	182.76 ± 4.87
FLS	1.84 ± 0.21	8.91 ± 0.12	26.22 ± 33	280.99 ± 10.36	183.38 ± 6.83
KCM	4.56 ± 0.26	9.19 ± 0.07	25.76 ± 20	261.74 ± 6.78	105.09 ± 4.03
Tap water	4.08 ± 0.27	7.73 ± 0.10	25.52 ± 36	263.26 ± 11.69	173.94 ± 7.50

*R. Kyambura* River Kyambura

*KCB* Kazinga channel bank

*KCM* Kazinga channel mid

*FLS* Fish landing site

### Correlation between waterborne parasites and physical parameters

Linear correlation analysis showed a strong inverse correlation between the presence of *Acanthamoeba* spp., *Hartmannella* spp., other FLA, *Cryptosporidium* spp. and *Giardia* spp. with DO (*P* < 0.05). The presence of these parasites showed a weak negative correlation with temperature. The presence of *Cryptosporidium spp.* showed a significant positive correlation (*P* < 0.05) with conductivity, pH and TDS. Similarly, the presence of *Giardia* spp. showed a strong positive correlation with TDS. Detailed analysis is presented in Table 3.

**Table 3:** Correlation between physical parameters and waterborne diseases (*r* values at 95 % *CI*)

In situ Parameter	*Acanthamoeba* spp.	*Hartmannella* spp.	*Naegleria* spp.	Other amoeba	*Cryptosporidium* spp.	*Giardia* spp.
DO(mg/L)	-0.231^**^	-0.129^**^	0.019	-0.271^**^	-319^**^	-0.147^**^
Temp(°C)	-0.051	-0.131	0.089	- 0.066	-0.084	-0.072
Cond(μS/m)	0.09	0.09	-0.100^*^	0.154^**^	0.204^**^	0.105^*^
pH	0.05	0.08	0.075	0.084	0.181^**^	0.094
TDS(g/L)	0.098^*^	0.101	-0.109	0.148^**^	0.210^**^	0.142^**^

^*^ *P* < 0.05; ^**^ *P* < 0.01 (significant at these levels)

### Molecular identification of the FLA isolates and phylogenetic analysis

**Fig. 3:**
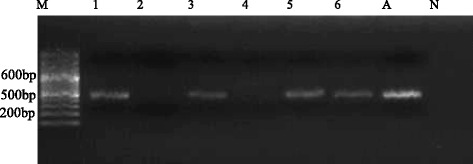
Agarose electrophoresis (2.5 %) showing amplification of JDP-PCR of *Acanthamoeba*. Lane M = DNA Ladder (100 bp), Lane A = Positive control, Lane N = Negative control, Lanes 1, 3, 5 & 6 = *Acanthamoeba* positive PCR product from obtained water samples

**Fig. 4:**
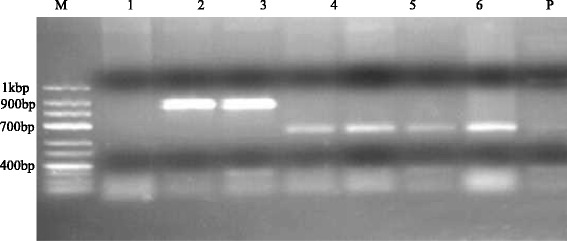
Agarose electrophoresis (2.5 %), showing amplification of CRN5/1137- PCR. M = DNA Ladder (100 bp), P = Positive control, N = Negative control, 1-6 = PCR products from obtained water samples

**Fig. 5:**
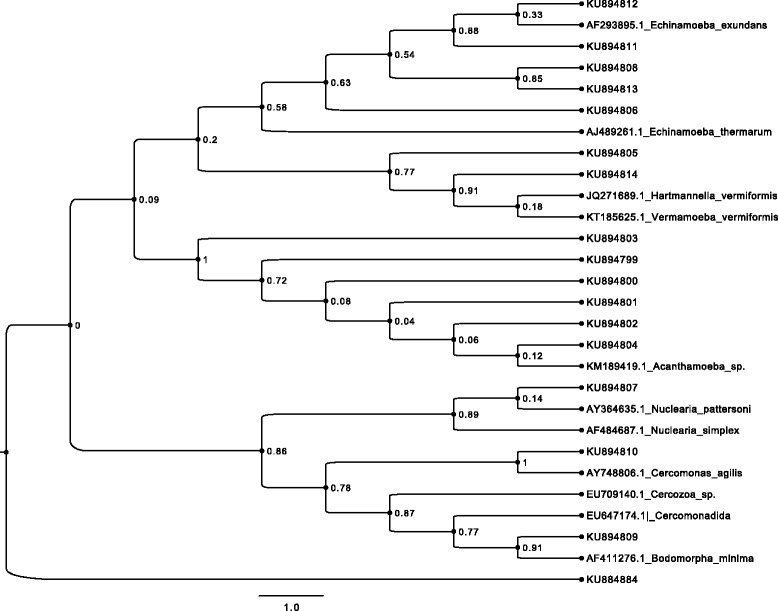
Phylogenetic tree showing the divergence of FLA. Comparison of GenBank sequences with their accession numbers (GenBank NCBI)

Thirty-one representative samples scored positive with PCR, 10 with JDP-PCR and 20 with CRN5/1137-PCR. The FLA isolates that produced the strongest positive bands (Figs. 3 and 4) were further sequenced and investigated by phylogenetic analysis. Following sequence blasting and comparison with the GenBank results from NCBI (Fig. 5, Table 4 and Appendix: Table 6), the following species was identified: *Acanthamoeba* spp., *Acanthamoeba polyphaga, Hartmannella vermiformis*, *Nuclearia pattersoni*, *Echinamoeba exundans*, *Bodomorpha minima* and *Cercomonas agilis*. The *Acanthamoeba* sequences got belonged to the group of sequence types T1, T4, and T11. All the parasites identified in this study were matched with the reported diseases they cause in humans (Table 5).

**Table 4:**
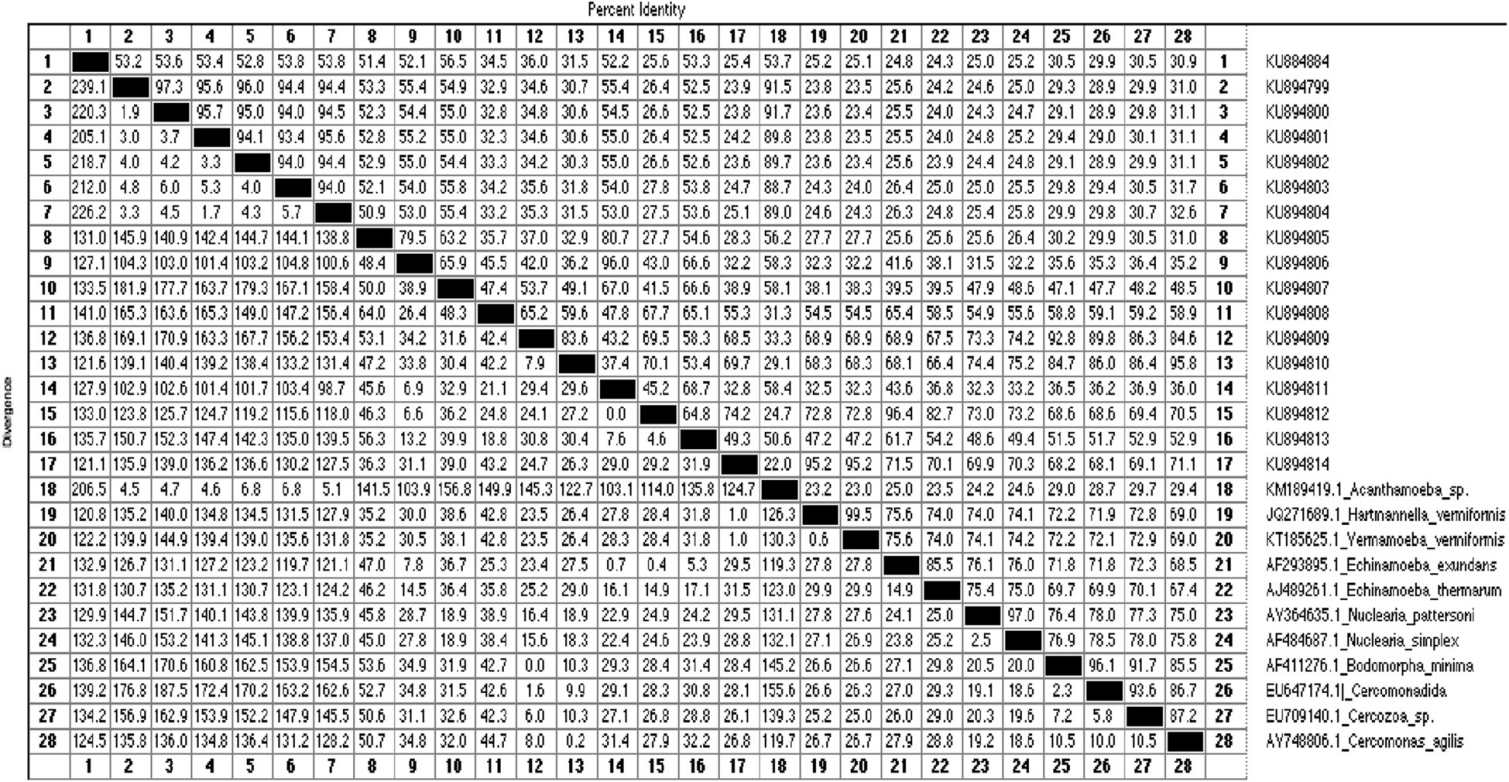
Estimation of evolutionary divergence between sequences

**Table 5:** Parasites isolated and associated human diseases

NCBI Accession No.	Genus and species	Associated Diseases
KU884884	*Acanthamoeba* spp. (T1)	Encephalitis [11, 48]
KU894799	*Acanthamoeba* spp. (T4)	Keratitis [48–50]
KU894800	*Acanthamoeba* spp. (T4)	Keratitis [48–50]
KU894801	*Acanthamoeba polyphaga.*(T4)	Keratitis [48–50]
KU894802	*Acanthamoeba* spp. (T4)	Keratitis [48–50]
KU894803	*Acanthamoeba* spp. (T4)	Keratitis [48–50]
KU894804	*Acanthamoeba* spp. (T11)	Keratitis [48–50]
KU894805	*Hartmanella vermiformis*	Keratitis [14, 41, 42]
KU894806	*Echinamoeba exundans*	Keratitis [42, 44]
KU894807	*Nuclearia pattersoni*	Unknown
KU894808	*Echinamoeba exundans*	Unknown
KU894809	*Bodomorpha minima*	Unknown
KU894810	*Cercomonas agilis*	Unknown
KU894811	*Echinamoeba exundans*	Unknown
KU894812	*Echinamoeba exundans*	Unknown
KU894813	*Echinamoeba exundans*	Unknown
KU894814	*Hartmanella vermiformis*	Unknown
-	*Cryptosporidium* spp.	Cryptosporidiosis [18, 51]
-	*Giardia* spp.	Giardiasis [10, 18]

## Discussion

The availability of potable water in poor countries is a challenge as it causes serious health problems. Polluted water sources are the major causes for the prevalence of waterborne infections which sometimes results in severe morbidities and mortalities [3, 28]. Diseases such as cholera, campylobacteriosis, shigellosis, salmonellosis and a variety of fungal and parasitic infections are known to affect a number of rural communities in sub-Saharan Africa [29], but infections due to FLA, *Cryptosporidium* and *Giardia* due to contaminated water sources are less reported. Acanthamoebiasis (brain, eye and skin infection), Hartmannellosis (respiratory tract infections or generalised fatal meningoencephalitis) and Naegleriasis (brain-eating amoeba infection) are not mentioned as possible infectious diseases in Uganda, and yet they may be silently occurring among individuals causing severe pathogenic effects. Cryptosporidiosis and Giardiasis which are diseases of poverty have over-time been neglected. They are known to be prevalent among communities which lack access to clean potable water supply [7, 9, 30]. Most communities in QEPA, like any other protected wildlife areas in Africa, are surrounded by poor communities with an average income of less than US$1 per person per day [31]. This level of income limits the communities from accessing privately owned water resources that provide safe water in the area as the price for this essential services is costly.

In the present study, all parasites except *Hartmannella* spp*.* and *Naegleria* spp. were more prevalent in the rainy season than the dry season, possibly due to contamination of the water sources with sewage or faeces through flooding. Previous studies have indicated that most waterborne parasites, especially FLA are more prevalent in cold rainy months of the year [32], whereas some like *Hartmannella* spp and *Naegleria* spp. are more common in warmer months [33]. Tap water had a higher prevalence of FLA than the natural water source. Considering specific natural water sites, prevalence and mean numbers of FLA were higher in KCB, FLS and R. Kyambura compared to KCM. These high numbers could be explained by high TDS due to organic matter from rotting leaves, animal and human faeces which are from the run-off from the land. Water from KCM appeared clear with less organic matter, the reason for few parasite presence. Tap water, on the other hand, had more FLA prevalence likely because all taps are supplied by the same natural water source with high TDS and organic matter, consequently, resulting in more biofilm formation along the piped water network. This is consistent with findings from other studies which explain that microorganisms settle on the inner surfaces of water pipes later becoming a source of secondary microbial contamination [34]. *Acanthamoeba*, *Hartmannella*, *Naegleria* and other FLA are known to thrive in areas containing high bacterial content which provide them with nutrition [11, 35]. The prevalence of *Cryptosporidium* and *Giardia* in these water sources were not significant but their mere presence is a health concern. The presence of *Cryptosporidium* and *Giardia* is due to human and animal faecal contamination of the water sources. Water sources for the communities in QEPA are all exposed to contamination from various points such as (1) hotels whose drainage enters directly into the water bodies, (2) fishing communities living nearby using shallow latrines from which excreta goes directly into the water bodies, (3) wild and domestic animal excreta also directly or indirectly ending up into water used by communities, and (4) Domestic refuse, rotten plants and soil entering into the water.

The mean DO (mg/L) at all the water sources did not meet the minimal requirement of 4.90 mg/L set by Uganda National Bureau of Standards (UNBS). Reduced DO is due to higher levels of TDS in the water. Decreased DO levels can be indicative of a large number bacteria and an excess amount of biological oxygen demand (BOD) due to untreated sewage, organic discharges and anoxic discharges, which deplete DO [36, 37]. Total Dissolved Solids are known to facilitates parasite proliferation [17, 38]. Linear correlation analysis showed that DO level had a negative correlation with the presence of *Acanthamoeba* spp., *Hartmannella* spp., other FLA, *Cryptosporidium* spp. and *Giardia* spp*.* Sometimes more parasites were found at slightly lower DO and less at higher DO. Previous studies have demonstrated that FLA, *Cryptosporidium* and *Giardia* can survive at low levels of DO [39]. In general, these parasites are reported to be more common in organic matter contaminated, bacteria-rich water irrespective of the physical parameters of the water [32]. However, often, they may be influenced by fluctuations in different physical parameters [40].

This study found pathogenic and non-pathogenic FLA in the water samples from the study sites. The potentially pathogenic FLA identified was *Acanthamoeba* spp. (T1, T4 and T11), *A. polyphaga* and *H. vermiformis. A. polyphaga* is one of the many *Acanthamoeba* spp. that cause GAE, AK, sinusitis and cutaneous infections especially in HIV/AIDS patients and other immunocompromised individuals [13, 41]. The inclusion of *H. vermiformis* on the list of human parasites was previously challenged by scientists citing lack of substantial evidence. However, recently *H. vermiformis* has consistently been mentioned as possible human parasites following its isolation in various mixed human AK infections [14, 41, 42] and as a result of their experimental effects in animal models [43]. A case of mixed keratitis infection reported due to *H. vermiformis* [14, 41] and another recent report regarding *Vahlkampfia avara* in Iran, in a mixed infection with *Acanthamoeba* spp. [44] should place more attention on other FLA not originally considered pathogenic. This means that it is possible for more FLA found in water, soil, dust, and other areas to become virulent anytime, given a conducive environment.

*C. parvum* and *G. lamblia* are the most common waterborne pathogens associated with diarrhoea in developing countries [45] and yet there is little concern by authorities in mitigating their effects. The presence of FLA, *Cryptosporidium* and *Giardia* in all the water sources in QEPA possess a high risk to the communities. In QEPA, water used by households, park staff, and restaurants comes from River Kyambura, Kazinga channel, Lake George and Edward, where most wild animals inhabit or water themselves. All the human communities living at the periphery of QEPA have their homes located close to the water bodies from which they fetch water for domestic use. During the rainy season, wash off of human and animal faecal matter from land into the water bodies is severe, with an eventual concentration of a large portion of the organic matter at points where communities fetch water from. This, coupled with inadequate water treatment, poor hygiene practices (irregular washing of water vessels), drinking unboiled water and ignorance, predisposes community members to a variety of parasites. Most water parasites are highly abundant during the rainy seasons [46] with a resultant concentration of parasites to points where animals and humans have access to water, dictating a continuous pattern of infection. The QEPA communities whose population is increasing at an alarming rate continues to compete for the same resources with the wildlife. They use the same water for their livestock and families. Sharing of this resource by wildlife and domestic animals with no definitive disease control measures exposes them to a continued cycle of infection. The fact that certain potentially pathogenic waterborne parasites like FLA, *Cryptosporidium* and *Giardia* are not often mentioned as possible infectious agents easily transmitted from water and contaminated food poses a risk of silent infections and death [47].

High-level community awareness, policy formulations and regular surveillance is needed in order to mitigate the pathogenic effects of waterborne parasites. This can be possible through “One Health” initiatives involving multidisciplinary teams of animal health workers, medical workers, politicians, quality control officers, local key informants and opinion leaders sharing ideas on the best way forward in improving water safety.

## Conclusion

The study shows that the protozoan parasites such as FLA, *Cryptosporidium* spp. and *Giardia* spp. found in the water resources in QEPA are a public health concern. This evidence shows the need for the relevant authorities in Uganda to initiate programmes for the control and prevention of diseases caused by these parasites. There must be a commitment from the government to supply clean potable water to the communities and this initiative must be of high priority. Community programmes must be initiated to educate the people on water safety measures, personal hygiene and water treatment processes. This study will, therefore, help in engaging the government in providing the necessary resources to improve community livelihoods of QEPA communities.

## Abbreviations

+ve, Positive; °C, Celsius; AK, amoebic keratitis; CNS, Central Nervous System; FLA, free-living amoeba; FLS, fish landing site; g, Grams; *g*, *gravitational force*; GAE, granulomatous amoebic encephalitis; HIV/AIDS, Human Immunodeficiency Virus/Acquired Immune Deficiency Syndrome; KCB, Kazinga channel bank; KCM, Kazinga channel mid; L, Liter; mg, Milligrams; ml, Milliliters; mM, millimolar; NCBI, National Center for Biotechnology Information; No., Number; PA, Protected Area; PAM, Primary amoebic meningoencephalitis; pg, picogram; pmol, picomoles; Prev, Prevalence; QEPA, Queen Elizabeth Protected Area; rpm, revolutions per minute; UNESCO, United Nations Educational, Scientific and Cultural Organization; V, Volts; μS, Microseconds.

## Appendix

**Table 6:** Given Accession Numbers from NCBI and Species from the Blast

Given NCBI Accession No.	Sequence_code	Species from Blast	Accession No.	Query coverage (%)	E-value	Identity (%)
KU884884	JDP_1	Acanthamoeba sp.	KM189419.1	89	0	98
KU894799	JDP_a	Acanthamoeba sp.	KF733221.1	99	0	98
KU894800	JDP_b	Acanthamoeba sp.	KF928953.1	99	0	99
KU894801	JDP_c	Acanthamoeba polyphaga	DQ013363.1	99	0	97
KU894802	JDP_g	Acanthamoeba sp.	KR259814.1	91	0	100
KU894803	JDP_h	Acanthamoeba sp.	FJ422510.1	91	0	97
KU894804	JDPi	Acanthamoeba sp.	JX507295.1	93	0	99
KU894805	CRN5_c	Hartmanella vermiformis	JQ271689.1	56.00	0	84.00
		Vermamoeba vermiformis	KT185625.1	56.00	0	84.00
KU894806	CRN5_d	Echinamoeba exundans	AF293895.1	97.00	0	93.00
		Echinamoeba thermarum	AJ489261.1	97.00	0	85.00
		Medicopsis romeroi	KF015650.1	97.00	0	78.00
		Erysiphe mori	AB033484.2	97.00	0	78.00
		Massaria platanoidea	HQ599457.1	100.00	3.00E-58	77.00
		Trematosphaeria biappendiculata	GU205254.1	97.00	3.00E-58	78.00
		Lepidospaeria nicotiae	DQ384068.1	97.00	3.00E-58	78.00
		Verruculina enalia	AF053730.1	97.00	3.00E-58	78.00
		Ophioceras venezuelense	AF050476.1	97.00	3.00E-58	78.00
		Helicascus kanaloanus	AF053729.1	97.00	3.00E-58	78.00
KU894807	CRN5_e	Nuclearia pattersoni	AY364635.1	99	6.00E-88	77
		Nuclearia simplex	AF484687.1	99	6.00E-88	77
		Paraphysoderma sedebokerense	KJ563218.1	99	2.00E-74	74
		Archaeospora schenckii	FR773150.1	99	2.00E-74	74
		Blastocladiales sp	EF565163.1	99	2.00E-74	74
		Chlamydomonas debaryana	HQ662272.1	99	3.00E-73	74
		Phlyctochytrium palustre	JQ014022.1	99	4.00E-71	74
		Spizellomyces sp	AB586079.1	99	4.00E-71	74
		Funneliformis mossease	FR750227.1	99	5.00E-70	74
		Glomus sp	AJ301864.1	99	6.00E-69	74
KU894808	CRN5_g	Echinamoeba exundans	AF293895.1	96	0	75
		Echinamoeba thermarum	AJ489261.1	96	8.00E-70	68
		Nuclearia sp	LN875111.1	97	2.00E-57	67
		Entrophospora infrequens	FR865452.1	96	4.00E-54	68
		Spongilla lacustris	KC902349.1	96	9.00E-50	66
		Tremella globispora	U00976.1	92	3.00E-49	67
		Baikalospongia intermedia	EF095188.1	96	5.00E-47	66
		Glomus sp	AJ301864.1	96	2.00E-46	66
		Solmundella bitentaculata	EU247812.1	96	9.00E-44	66
KU894809	CRN5_h	Bodomorpha minima	AF411276.1	100	0	99
		Cercomonadida	EU647174.1	99	0	96
		Heteromita sp	AY905499.1	97	0	94
		Eimeriidae	EF024879.1	99	0	94
		Orciraptor agilis	KF207875.1	99	0	93
		Cercozoa sp	EU709140.1	98	0	93
KU894810	CRN5_i	Cercomonas agilis	AY748806.1	98	0	96
		Cercozoa sp	EU709140.1	96	0	95
		Orciraptor agilis	KF207875.1	89	0	91
		Eimeriidae	EF024879.1	89	0	90
		Viridiraptor invadens	KF207870.1	89	0	90
		Cercomonadida	EU647174.1	99	0	87
		Heteromita sp	AY905499.1	89	0	89
		Bodomorpha sp	HM536170.1	91	0	88
		Thaumatomonadida	EF024516.1	89	0	88
		Ebria tripartita	DQ303923.1	99	0	86
		Eocercomonas sp	HM536154.1	83	0	91
		Apiaceae	EF024041.1	82	0	91
KU894811	CRN5_k	Echinamoeba exundans	AF293895.1	98	0	99
		Echinamoeba thermarum	AJ489261.1	98	2.00E-80	83
		Eimeriidae	EF024879.1	98	2.00E-59	79
		Nucleariidae	EF024271.1	98	2.00E-59	79
		Rhizophydium sp	DQ536492.1	98	2.00E-59	78
		Chlamydomonas sp	AY220092.1	98	2.00E-59	78
		Leveillula taurica	AB033479.1	98	9.00E-59	78
KU894812	CRN5_l	Echinamoeba exundans	AF293895.1	99	0	99
		Echinamoeba thermarum	AJ489261.1	99	0	82
		Claroideoglomus lamellosum	FR773152.1	78	0	85
		Glomus sp	AJ301864.1	78	0	85
		Entrophospora infrequens	FR865452.1	78	0	85
		Cryptococcus sp	KM587000.1	82	0	84
		Filobasidium globisporum	AB075546.1	82	0	84
		Filobasidium uniguttulatum	AB032664.1	82	0	84
KU894813	CRN5_m	Echinamoeba exundans	AF293895.1	99	0	86
		Echinamoeba thermarum	AJ489261.1	86	0	77
		Claroideoglomus lamellosum	FR773152.1	86	0	74
		Glomus sp	AJ301864.1	86	0	74
		Entrophospora infrequens	FR865452.1	86	0	74
		Cyanophora paradoxa	FR865776.1	86	0	73
		Stylaster sp	EU645439.1	86	0	73
		Trechispora sp	AY803753.1	86	0	73
KU894814	CRN5_o	Hartmanella vermiformis	AY502960.1	100	0	99
		Vermamoeba vermiformis	KC161965.1	100	0	98

## Additional file

Additional file 1: Multilingual abstracts in the five official working languages of the United Nations. (PDF 566 kb)
