# Supplementary material for: Prevalence of pathogenic free-living amoeba and other protozoa in natural and communal piped tap water from Queen Elizabeth protected area, Uganda
Source: Infect Dis Poverty. 2016 Aug 3;5:68. doi: 10.1186/s40249-016-0162-5 (PMC4971666; doi:10.1186/s40249-016-0162-5)
Supplement: Additional file 1: — Multilingual abstracts in the five official working languages of the United Nations. (PDF 566 kb) [file 40249_2016_162_MOESM1_ESM.pdf]

Translation of the abstract into the five official working languages of the United Nations

انتشار الكائنات الدقيقة المسببة للأمراض كالأميبا وذوات الخلية الواحدة "البروتوزوا" في الانابيب الطبيعية و انابيب مياه الصنبور في منطقة المحمية الملكة اليزبيث , أوغندا

سيلسوس سينتى ,جوزيف ايرومة، ايرين نايغاغا، يوليوس مولیندوا، سيلفستر اوتشو، فيليب كيمودا ماغامبو , بينيغا غابربيلا نامارا، تشارلز دراغو كاتو، جورج ساباتيك ، كيفن مواونج، مايكل اوكادو.

الملخص

الخلفية: المياه تعيش فيها العوامل المسببة للمرض وحيدات الخلية "البروتوزوا" مثل الشوكمبية ,الهارتمانيلة , النيغلرية, الكريبتوسبوريديوم , و الجيارديا

كلها مسؤولة عن امراض مميتة وخاصة للأطفال وذوي الجهاز المناعي الضعيف, ولكن وجودها وانتشارها في بيئات معينة في الصحراء الافريقية الجنوبية الكبرى مازال غير معروفا لأغلب الباحثين ومسؤولي الصحة العامة و الأطباء

الغرض من هذه الدراسة هو تحديد وجود وانتشار العوامل المسببة للمرض والتي تعيش طليقة كالأميبا (FLA) , الكريبتوسبوريديوم والجيارديا في محمية منطقة الملكة اليزبيث (QEPA).

الطرق: تم جمع العيانات من الصنابير العامة ومواقع المياه الطبيعية في QEPA. تم قياس معلمات المياه العادية في الموقع. تم تفحص العينات للكشف عن وجود الطفيل الاميبا ,(FLA) بواسطة الزراعة ممحوضة ,ببيضيات الابواغ بواسطة تسلين-نلسن وخراجات الجيارديا بواسطة تقنية التعويم كبريتات الزنك . الطفيليات تم الكشف عنها مجهريا , تحديدها ,احصائها , من اجل الاميبا (FLA) تم استخراج الحمض النووي الدنا من اجل التكبير والتسلسل

النتائج: كلا من مصادر المياه الطبيعية و مياه الصنبور كانت ملوثة ب الأميبا (FLA), و الكريبتوسبوريديوم ,الجيارديا .كانت الكائنات الطفيلية اكثر وفرة في المواسم الباردة والممطرة ماعدا الهارمانيلا و النيغلرية , والتي كانت موجودة بكثرة في الاشهر الدافئة .انتشار الكائنات الطفيلية في مياه الصنابير اكثر منها في مصادر المياه الطبيعية. كان هناك ارتباط سلبي و قوي بين وجود الشوكمبية ,الهارتمانيلة , الكريبتوسبوريديوم والجيارديا مع الاكسجين الحال بالماء (P <0.05) (DO). وجود الابواغ الكريبتوسبوريديوم اعطت علاقة إيجابية قوية (P <0.05) مع الناقلية , درجة الحموضة والمواد الصلبة الذائبة (TDS) . في حيث وجود الجيارديا كان يعتمد على علاقة إيجابية قوية مع المواد الصلبة المذابة (TDS) . التنميط الجيني الجزيئي للأميبا (FLA) انتجت 7شوكمبيات , 5 أميبا ,2 الهارتمانيلة , 1بودومورفا , 1 نوكليريا و 1 المذيلة التسلسلات الجينية

الاستنتاج: تم الكشف في جميع مصادر المياه بانها ملوثة وتحتوي على العوامل الأولية المسببة للأمراض والتي يمكن ان تكون سببا لعدد من الحالات المرضية المميتة الصامتة لعديد من مرضى الاسر المحلية في (QEPA) وهذا يعني بان الماء المستخدم من قبل التجمعات السكنية في (QEPA) هو من النوعية الرديئة وهو يجعلهم عرضة للإصابة لعديد من العدوة والتي سببها وحيدات الخلية بما في ذلك الاميبا (FLA) والذين لم يكتب تقريرا بخصوص صحتهم العامة مما يستلزم اتخاذ تدابير وإجراءات مناسبة للحفاظ على معايير سلامة المياه.

Translated from English version into Arabic by Diana H, through

TRANSLATORS
WITHOUT BORDERS

乌干达伊丽莎白女王保护区的天然水和公共管道自来水中致病性自由生活阿米巴及其他病原菌的流行情况

Celsus Sente, Joseph Erume, Irene Naigaga, Julius Mulindwa, Sylvester Ochwo, Phillip Kimuda Magambo, Benigna Gabriela Namara, Charles Drago Kato, George Sebyatika, Kevin Muwonge, Michael Ocaido

摘要

引言：水生病原虫，如棘阿米巴属、哈氏阿米巴属、耐格里属、隐孢子虫属和贾第鞭毛虫属，常感染人体尤其是儿童和免疫力低下人群，造成致命性疾病。但很多研究人员、公共卫生官员及医生却不了解其在南非撒哈拉沙漠的出现和流行情况。本研究旨在确定伊丽莎白女王保护区(QEPA) 阿米巴虫属(FLA)，隐孢子虫属和贾第鞭毛虫属的存在和流行情况。

方法：样本取自 QEPA 的供水管道和天然水。现场测量水样的物理参数。处理样本并通过无菌培养来检测 FLA 滋养体的出现，使用抗酸染色检测隐孢子虫卵囊，使用硫酸锌浮选技术检测贾第鞭毛虫囊肿。通过显微观察、识别、计数和记录寄生虫。提取 FLA 的基因组 DNA 用于扩增和测序。

结果：检测结果表明，天然和自来水水源均被 FLA、隐孢子虫属和贾地鞭毛虫属污染。除了哈氏阿米巴属、耐格里属在较暖的月份繁殖外，其他原生寄生虫在较冷的雨季繁殖能力更强。所有寄生虫在自来水中的流行率均比在天然水样中的高。溶解氧(DO) (P<0.05)与棘阿米巴属、哈氏阿米巴属，隐孢子虫属和贾第鞭毛虫属呈强负相关。隐孢子虫属的出现与电导率、pH 值和总溶解固体（TDS）呈显著正相关（P <0.05），而

贾第鞭毛虫属只与 TDS 呈强正相关。 FLA 的分子基因型产生 7 种棘阿米巴，5 种刺变虫属，2 种哈氏阿米巴属，1 种异养型鞭毛虫属，1 Nucleari 和 1 种单鞭滴虫属的部分序列。
结论：研究发现，QEPA 所有水样收集点均被病原虫污染，这可能是一系列农村家庭中不明发病和死亡的原因。研究表明， QEPA 社区所用水质较差，易造成多种包括 FLA 在内的病原虫感染，而从未报道过其对公共卫生重要影响。因此，QEPA 需要采用适当的水源安全措施。

Translated from English version into Chinese by Chen Jin, through

Prévalence des amibes libres et autres protozoaires pathogènes dans les eaux naturelles et l'eau du robinet de la zone protégée Queen Elizabeth en Uganda

Celsus Sente, Joseph Erume, Irene Naigaga, Julius Mulindwa, Sylvester Ochwo, Phillip Kimuda Magambo, Benigna Gabriela Namara, Charles Drago Kato, George Sebyatika, Kevin Muwonge, Michael Ocaido

Résumé

Contexte : Les protozoaires pathogènes aquatiques tels qu'*Acanthamoeba* spp., *Hartmannella* spp., *Naegleria* spp., *Cryptosporidium* spp. et *Giardia* spp. sont souvent responsables de maladies dévastatrices, notamment chez les enfants et les individus immunodéprimés. Pourtant, leur présence et leur prévalence dans certains environnements d'Afrique subsaharienne sont encore inconnus de la plupart des chercheurs, des services de santé publique et des médecins. L'objectif de cette étude était d'évaluer la présence et la prévalence des amibes libres, de *Cryptosporidium* et de *Giardia* dans la zone protégée Queen Elizabeth (QEPA).
Méthodes : Des échantillons ont été prélevés aux robinets communaux et dans des points d'eau naturels dans la QEPA. Les paramètres physiques de l'eau ont été mesurés *in situ*. Les échantillons ont été préparés pour détecter la présence de trophozoïtes d'amibes libres sur culture xénique, des oocystes de *Cryptosporidium* par coloration de Ziehl-Neelsen et des cystes de *Giardia* par technique de flottation au sulfate de zinc. Les parasites ont été observés au microscope, identifiés, comptés et enregistrés. Pour les amibes libres, l'ADN génomique a été extrait pour l'amplifier et le séquencer.
Résultats : Les eaux naturelles et du robinet étaient contaminées par des amibes libres, *Cryptosporidium* spp. et *Giardia* spp. Tous les protozoaires parasites étaient plus abondants durant la saison des pluies plus froide, sauf *Hartmannella* spp. et *Naegleria* spp. qui étaient plus présentes pendant les mois chauds. La prévalence des parasites était globalement plus élevée dans les échantillons d'eau du robinet que dans les eaux naturelles. Il y avait une forte corrélation négative entre la présence d'*Acanthamoeba* spp., *Hartmannella* spp., *Cryptosporidium* spp. et *Giardia* spp. et l'oxygène dissous (P<0,05). La présence de *Cryptosporidium* spp. a montré une corrélation positive significative (P<0,05) avec la conductivité, le pH et les matières dissoutes totales. La présence de *Giardia* spp. n'avait qu'une corrélation positive forte avec les matières dissoutes totales. Le génotypage moléculaire des amibes libres a produit 7 séquences partielles d'*Acanthamoeba*, 5 d'*Echinamoeba*, 2 de *Hartmannella*, 1 de *Bodomorpha*, 1 de *Nuclearia* et 1 de *Cercomonas*.
Conclusions : Tous les sites de prélèvement d'eau se sont avérés contaminés par des protozoaires pathogènes, qui peuvent être la cause d'un grand nombre de maladies méconnues et de décès dans les foyers ruraux de la QEPA. Ceci implique que l'eau utilisée par les populations de la QEPA est de mauvaise qualité et expose les habitants à des infections par différents protozoaires, notamment des amibes libres, dont l'importance en santé publique n'a jamais été rapportée et qui nécessitent l'adoption de mesures adaptées d'assainissement de l'eau.

Translated from English version into French by Suzanne Assenat, through

Распространенность свободно живущих патогенных амёб и других простейших в природных водоемах и коммунальной водопроводной воде в заповедном районе имени королевы Елизаветы в Уганде

Celsus Sente, Joseph Erume, Irene Naigaga, Julius Mulindwa, Sylvester Ochwo, Phillip Kimuda Magambo, Benigna Gabriela Namara, Charles Drago Kato, George Sebyatika, Kevin Muwonge, Michael Ocaido

Аннотация

Краткая информация: Патогенные простейшие, обитающие в воде, такие как *Acanthamoeba* spp., *Hartmannella* spp., *Naegleria* spp., *Cryptosporidium* spp. *и Giardia* spp., часто вызывают опасные болезни, особенно у детей и лиц с ослабленным иммунитетом, однако их наличие и распространенность в определенной среде в странах Африки к югу от Сахары до сих пор неизвестна большинству исследователей, работников общественного здравоохранения и медицинских работников. Цель данного исследования состояла в том, чтобы выявить наличие и распространенность свободно живущих патогенных амёб (СПА), *Cryptosporidium* и *Giardia*, в заповедном районе имени королевы Елизаветы (QEPA).

Методы: Пробы воды брали из водопроводных кранов и природных водных объектов в QEPA. Физические параметры воды измерялись на местах. Образцы обрабатывались с целью обнаружения присутствия трофозоитов СПА путем ксеникальной культивации, ооцисты *Cryptosporidium* выявляли путем окраски по Цилю-Нильсену, а кист *Giardia* выявляли методом флотации сульфата цинка. Паразитов наблюдали при помощи микроскопа, определяли, подсчитывали и записывали. Геномную ДНК СПА извлекали для амплификации и секвенирования.

Результаты: Как природные, так и водопроводные источники воды были заражены СПА, *Cryptosporidium* spp. и *Giardia* spp. Все простейшие паразиты были более распространены в холодный сезон дождей, за исключением *Harmannella* spp. и *Naegleria* spp., которые встречались чаще в теплое время года. Распространенность всех паразитов в водопроводной воде была выше, чем в пробах воды из природных водоемов. Наблюдалась значительная отрицательная корреляция между присутствием *Acanthamoeba* spp., *Hartmannella* spp., *Cryptosporidium* spp. и *Giardia* spp. и растворенным кислородом (РО) (P<0,05). Наличие *Cryptosporidium* spp. показывало значимую положительную корреляцию (P <0,05) с проводимостью, pH и общим содержанием растворенных веществ (ОРВ); в то время как наличие *Giardia* spp. имело лишь значительную положительную корреляцию с ОРВ. Молекулярное генотипирование СПА показало следующие частичные последовательности: 7 *Acanthamoeba,* 5 *Echinamoeba,* 2 *Hartmannella,* 1 *Bodomorpha*, 1 *Nuclearia* и 1 *Cercomonas*.

Выводы: Оказалось, что все места забора проб воды были загрязнены патогенными простейшими, которые могли быть причиной целого ряда бессимптомных заболеваний, в том числе со смертельным исходом, среди сельских домохозяйств в QEPA. Это означает, что вода, используемая общинами в QEPA, плохого качества и предрасполагает к различным протозойным инфекциям, включая СПА, значение которых для общественного здравоохранения до сих пор не констатировалось, что обусловливает необходимость принятия надлежащих мер по обеспечению безопасности воды.

Translated from English version into Russian by Oksana Weiss, through

TRANSLATORS WITHOUT BORDERS

Prevalencia de la ameba patógena de vida libre y otros protozoos en el agua natural y en el agua de grifo comunitario dentro del Área Protegida Reina Elizabeth en Uganda.

Celsus Sente, Joseph Erume, Irene Naigaga, Julius Mulindwa, Sylvester Ochwo, Phillip Kimuda Magambo, Benigna Gabriela Namara, Charles Drago Kato, George Sebyatika, Kevin Muwonge, Michael Ocaido

Resumen

Antecedentes: Los protozoos patógenos que habitan en el agua, como el *Acanthamoeba* spp., *Hartmannella* spp., *Naegleria* spp., *Cryptosporidium* spp. *y Giardia* spp. son generalmente responsables de enfermedades devastadoras, particularmente en niños y en personas inmunodeprimidas. Sin embargo, la mayoría de los investigadores, oficiales de salud pública y médicos desconocen su presencia y prevalencia en ciertos entornos en África subsahariana. El objetivo del presente estudio fue establecer la presencia y prevalencia de amebas patógenas de vida libre (FLA) *Cryptosporidium* and *Giardia* en el Área Protegida Reina Elizabeth (QEPA).

Métodos: Se recolectaron muestras de sitios de agua natural y de grifos comunitarios en QEPA. Los parámetros físicos del agua se midieron *in situ*. Se procesaron las muestras para detectar la presencia de trofozoíto de ameba

patógena de vida libre por cultivo xénico, oocistos de *Cryptosporidium* por tinción de Ziehl-Neelsen y quistes de *Giardia* por la técnica de flotación con sulfato de zinc. Los parásitos se observaron por microscopio, se los identificó, contó y registró. Para las amebas patógenas de vida libre se extrajo el ADN genómico para amplificación y secuenciación.

Resultados: Tanto las fuentes de agua natural como las de los grifos estaban contaminadas con amebas patógenas de vida libre, *Cryptosporidium* spp. y *Giardia* spp. Todos los protozoos parásitos eran más abundantes en la temporada de lluvia, que es más fría, con la excepción de *Harmannella* spp. y *Naegleria* spp. que se daban más en los meses más cálidos. La prevalencia de todos los parásitos fue más elevada en las muestras de agua de grifo que en las muestras de agua natural. Se observó una fuerte correlación negativa entre la presencia de *Acanthamoeba* spp., *Hartmannella* spp., *Cryptosporidium* spp. y *Giardia* spp. con Oxígeno Disuelto (OD) (P<0,05). La presencia de *Cryptosporidium* spp. mostró una correlación significativamente positiva (P<0.05) con la conductividad, pH y el total de sólidos disueltos (TDS); mientras que la presencia de *Giardia* spp. solo tenía una fuerte correlación positiva con los TDS. La genotipificación molecular de las amebas patógenas de vida libre produjeron secuencias parciales 7 *Acanthamoeba,* 5 *Echinamoeba,* 2 *Hartmannella,* 1 *Bodomorpha*, 1 *Nuclearia* y 1 *Cercomonas*.

Conclusiones: Se encontró que todos los sitios de recolección de agua estaban contaminados con protozoos patógenos que podrían posiblemente producir una cierta cantidad de morbilidad silenciosa y mortalidad en viviendas rurales en QEPA. Esto implica que el agua que utilizan las comunidades en QEPA es de mala calidad y que los predispone a una variedad de infecciones por protozoos que incluyen la ameba patógena de vida libre cuya importancia para la salud pública nunca se reportó. Por lo tanto, se necesita de la adopción de medidas adecuadas de seguridad para el agua.

Translated from English version into Spanish by Maria Alejandra Aguada, through

TRANSLATORS
WITHOUT BORDERS
